# Supplementary material for: Incidence and Impacts of Inflammatory Bowel Diseases among Kidney Transplant Recipients: A Meta-Analysis
Source: Med Sci (Basel). 2020 Sep 16;8(3):39. doi: 10.3390/medsci8030039 (PMC7565568; doi:10.3390/medsci8030039)
Supplement: Supplementary file 1 [file medsci-08-00039-s001.pdf]

Search strategy for OVID MEDLINE database.

Step	Search Term
1	Inflammatory bowel diseases OR Crohn’s disease OR Ulcerative colitis
2	Graft failure OR graft loss OR graft survival OR mortality OR death OR remission OR outcome
3	Kidney transplant OR renal transplant
4	1 AND 2 AND 3

Search results: 200

Search strategy for EMBASE database.

('inflammatory bowel disease' OR 'Crohn disease' OR 'ulcerative colitis') AND ('kidney transplant' OR 'renal transplant' OR 'kidney transplantation' OR 'kidney graft' OR 'kidney graft rejection' OR 'patient history of kidney transplantation')

Search results: 627

Search strategy for Cochrane Library

(‘Inflammatory bowel diseases’ OR ‘Crohn’s disease’ OR ‘Ulcerative colitis’) AND (‘Graft failure’ OR ‘graft loss’ OR ‘graft survival’ OR ‘mortality’ OR ‘death’ OR ‘remission’ OR ‘outcome’) AND (‘Kidney transplant’ OR ‘renal transplant’)

Search results: 14

Figure S1. Risk of bias summary.

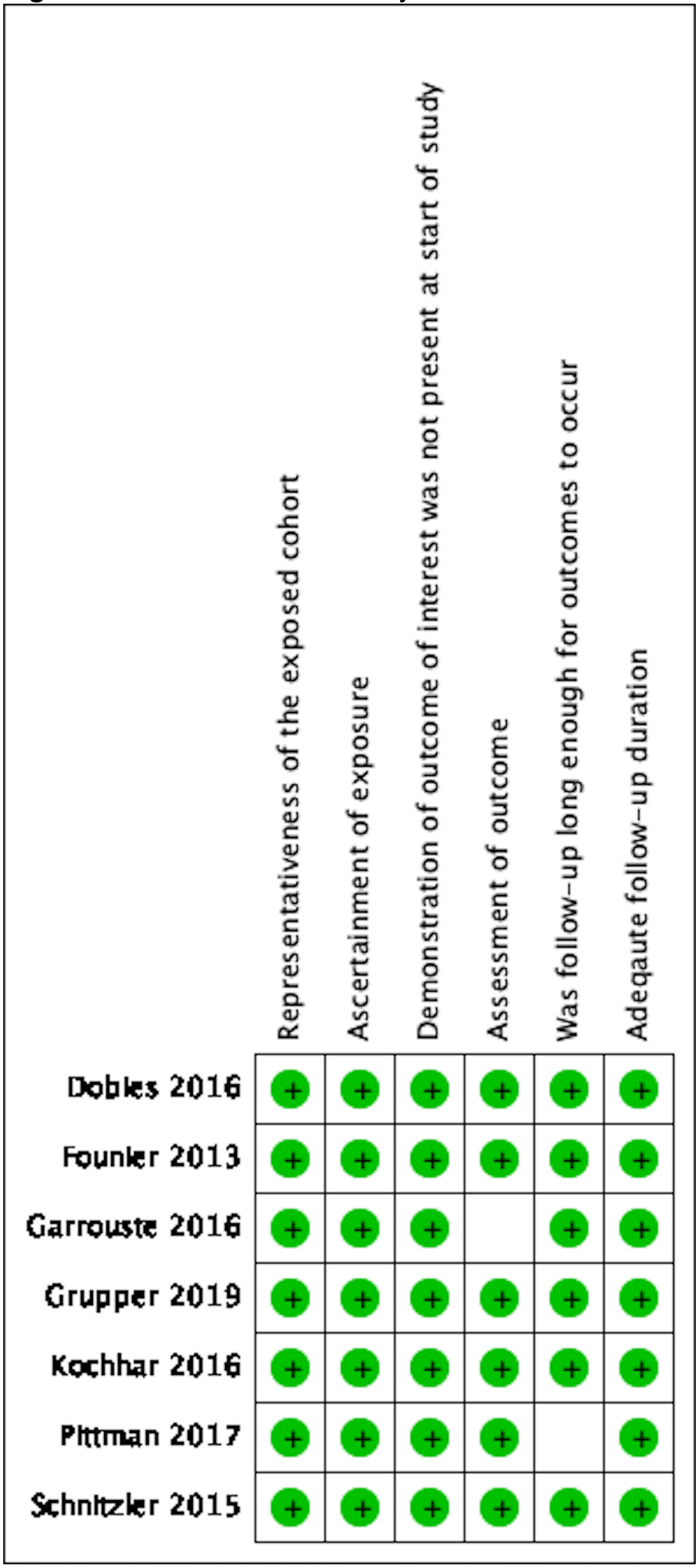

Main results for Model 1, Random effects (MM), Z-Distribution, Logit event rate

Covariate	Coefficient	Standard Error	95% Lower	95% Upper	Z-value	2-sided P-value
**Intercept**	0.2544	1.4027	-2.4949	3.0036	0.18	0.8561
**Age**	-0.0336	0.0307	-0.0937	0.0266	-1.09	0.2739

Statistics for Model 1

Test of the model: Simultaneous test that all coefficients (excluding intercept) are zero
Q = 1.20, df = 1, p = 0.2739
Goodness of fit: Test that unexplained variance is zero
Tau^2 = 0.0868, Tau = 0.2946, I^2 = 28.53%, Q = 7.00, df = 5, p = 0.2209

Comparison of Model 1 with the null model

Total between-study variance (intercept only)
Tau^2 = 0.1098, Tau = 0.3313, I^2 = 34.57%, Q = 9.17, df = 6, p = 0.1642
Proportion of total between-study variance explained by Model 1
R^2 analog = 0.21

Number of studies in the analysis 7

Main results for Model 1, Random effects (MM), Z-Distribution, Logit event rate

Covariate	Coefficient	Standard Error	95% Lower	95% Upper	Z-value	2-sided P-value
**Intercept**	-0.7514	0.5665	-1.8617	0.3588	-1.33	0.1847
**Male**	-1.1142	1.1425	-3.3534	1.1250	-0.98	0.3294

Statistics for Model 1

Test of the model: Simultaneous test that all coefficients (excluding intercept) are zero
Q = 0.95, df = 1, p = 0.3294
Goodness of fit: Test that unexplained variance is zero
Tau^2 = 0.0926, Tau = 0.3043, I^2 = 30.05%, Q = 7.15, df = 5, p = 0.2099

Comparison of Model 1 with the null model

Total between-study variance (intercept only)
Tau^2 = 0.1098, Tau = 0.3313, I^2 = 34.57%, Q = 9.17, df = 6, p = 0.1642
Proportion of total between-study variance explained by Model 1
R^2 analog = 0.16

Number of studies in the analysis 7

Main results for Model 1, Random effects (MM), Z-Distribution, Logit event rate

Covariate	Coefficient	Standard Error	95% Lower	95% Upper	Z-value	2-sided P-value
**Intercept**	-0.1107	0.8499	-1.7764	1.5550	-0.13	0.8963
**Follow-up**	-0.0170	0.0104	-0.0373	0.0033	-1.64	0.1010

Statistics for Model 1

Test of the model: Simultaneous test that all coefficients (excluding intercept) are zero

Q = 2.69, df = 1, p = 0.1010

Goodness of fit: Test that unexplained variance is zero

Tau^2 = 0.0600, Tau = 0.2449, I^2 = 16.70%, Q = 3.60, df = 3, p = 0.3079

Comparison of Model 1 with the null model

Total between-study variance (intercept only)

Tau^2 = 0.1946, Tau = 0.4411, I^2 = 43.02%, Q = 7.02, df = 4, p = 0.1348

Proportion of total between-study variance explained by Model 1

R^2 analog = 0.69

Number of studies in the analysis 5
